# Case Report: Uncommon presentation of Ewing sarcoma complicated by hemophagocytic lymphohistiocytosis: a diagnostic dilemma and therapeutic challenge

**DOI:** 10.3389/fonc.2025.1613906

**Published:** 2025-08-11

**Authors:** Zhuan Zou, Bin Chen, Fajuan Tang, Xihong Li, Chengshuang Huang

**Affiliations:** ^1^ Department of Emergency, West China Second University Hospital, Sichuan University, Chengdu, China; ^2^ Key Laboratory of Birth Defects and Related Diseases of Women and Children (Sichuan University), Ministry of Education, Chengdu, China

**Keywords:** hemophagocytic lymphohistiocytosis, extraosseous ewing sarcoma, case report, Epstein-Barr virus (EBV), malignancy-associated HLH

## Abstract

Hemophagocytic lymphohistiocytosis (HLH), also referred to as hemophagocytic syndrome (HPS), is a life-threatening condition characterized by excessive immune activation. It is commonly associated with genetic mutations, infections, autoimmune diseases, and malignancies. Malignancy-associated HLH (M-HLH) is most frequently observed in hematologic malignancies, such as lymphoma and leukemia, while its occurrence in solid tumors is exceedingly rare. Here, we report a unique case of temporal bone Ewing sarcoma complicated by HLH and Epstein-Barr virus (EBV) infection. Despite intensive chemotherapy, the patient ultimately succumbed to multi-organ failure and septic shock. To the best of our knowledge, this is the first documented case of Ewing sarcoma associated with HLH.

## Introduction

Hemophagocytic lymphohistiocytosis (HLH) is a rare but life-threatening inflammatory syndrome caused by excessive immune system activation. It is characterized by hyperactivation of immune cells, primarily T cells and macrophages, leading to phagocytosis of blood cells, including erythrocytes, leukocytes, and platelets. Clinically, HLH typically presents with persistent high fever, splenomegaly, pancytopenia, and elevated inflammatory markers such as serum ferritin (1, 2).

The condition can result in systemic inflammation, tissue damage, and multi-organ dysfunction. Based on genetic predisposition and underlying triggers, HLH is classified into primary HLH (P-HLH) and secondary HLH (S-HLH). P-HLH arises from congenital defects in lymphocyte cytotoxicity, often involving mutations in genes such as PRF1 and UNC13D, and is predominantly observed in pediatric patients. In contrast, S-HLH is triggered by systemic immune dysregulation secondary to malignancies, infections, or autoimmune diseases (3).

Notably, the incidence of malignancy-associated HLH (M-HLH) has been increasing, particularly in adult patients with hematologic malignancies such as lymphoma and acute leukemia. Despite its clinical significance as a life-threatening hyperinflammatory syndrome, M-HLH associated with solid tumors remains exceedingly rare (4). Among reported cases of M-HLH associated with solid tumors, common triggers include gastric cancer, glioblastoma, head and neck squamous cell carcinoma and and other tumors (5–9). Ewing sarcoma (ES) is a rare and aggressive malignant tumor that primarily arises in bone or soft tissue. To date, only a limited number of cases have documented the coexistence of HLH and ES. This unusual association underscores the need for further investigation into its underlying mechanisms.

Here, we present the case of a 7-year-and-8-month-old female who initially presented with a painless mass in the right temporal region. During the clinical course, she was sequentially diagnosed with HLH and primary ES of the temporal bone. Despite aggressive treatment, including intensive chemotherapy (IVDE regimen), immunomodulatory therapy, and anti-infective supportive care, the patient succumbed to gastrointestinal hemorrhage, septic shock, and multi-organ failure. This case highlights the diagnostic and therapeutic challenges associated with the rare coexistence of ES and HLH. By analyzing the clinical course and management of this patient, along with a systematic review of the literature, we aim to provide valuable insights into the diagnosis and treatment of this rare and challenging condition.

## Case description

A previously healthy 7-year-8-month-old girl presented in December 2018 with a six-month history of a painless right temporal mass. The mass had grown by over 20% in size during the past two months. This was accompanied by persistent high-grade fever (temperature >39.5°C) lasting 10 days. A prior biopsy of the facial mass at an outside hospital revealed inflammatory changes consistent with cellulitis. Despite receiving empirical antibiotic therapy, the lesion progressively enlarged, though the specific regimen remains unknown. Her vaccination history was up to date, with no family history of genetic bone or soft tissue disorders, and no familial predisposition to HLH.

**Figure 1:**
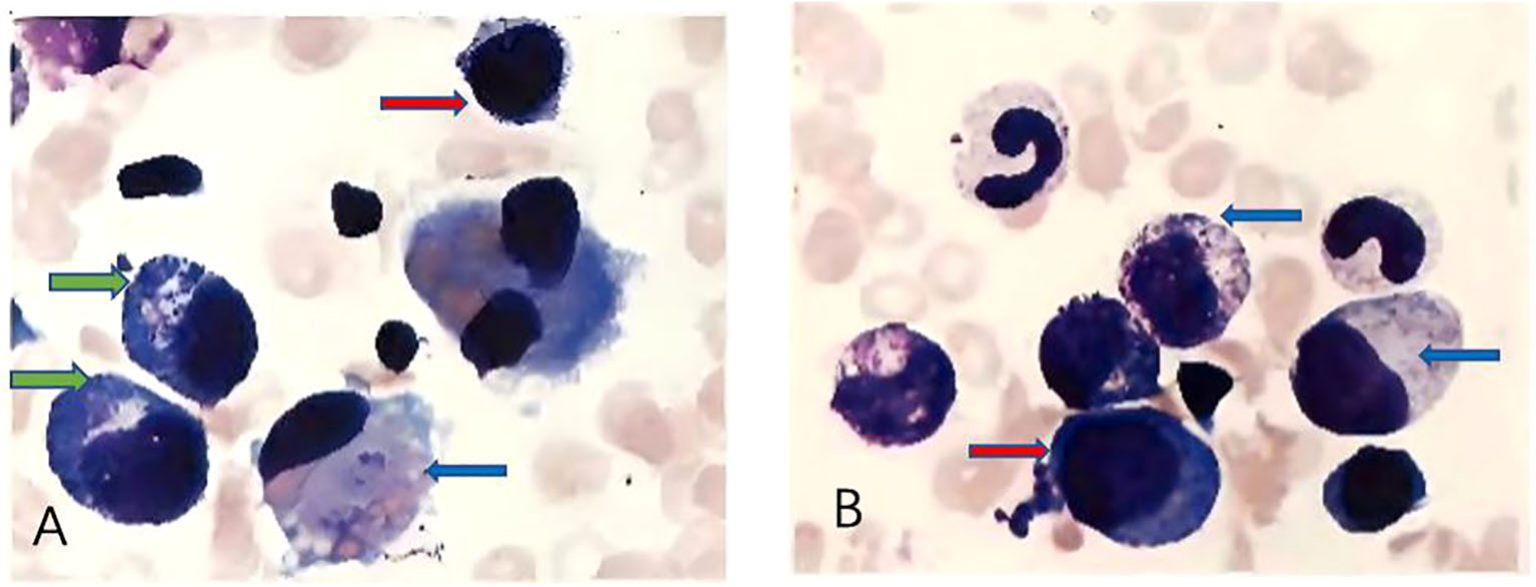
**(A, B)** show different microscopic fields from bone marrow cytology demonstrating hemophagocytosis. Red arrows indicate hemophagocytes, characterized by deep blue, round, or irregularly shaped cells. Blue arrows point to larger deep blue cells with pale intracellular areas, representing hemophagocytes actively engulfing erythrocytes. Green arrows highlight smaller, round, deep blue cells with irregular margins, which correspond to hemophagocytes phagocytosing platelets.

**Figure 2:**
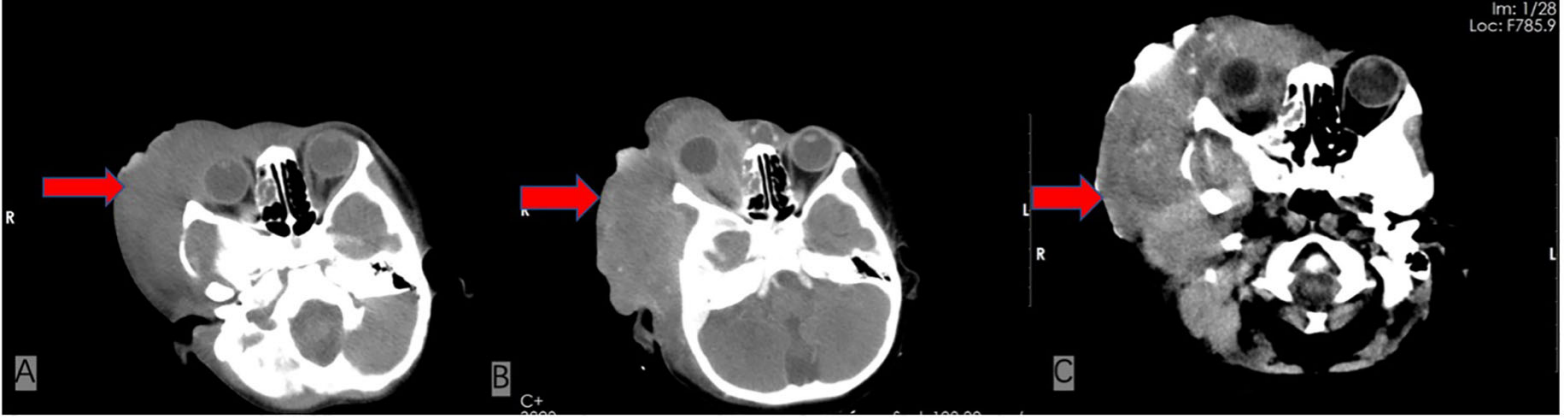
Comparison of serial cranial CT scans at the same axial level before and after treatment. **(A)** Contrast-enhanced CT from December 2018, at the time of admission, reveals a 4.6 cm × 11 cm × 11 cm mass in the right maxillofacial region with associated zygomatic arch bone destruction. **(B)** Follow-up CT in May 2019, after four cycles of chemotherapy, shows a newly developed metastatic lesion (4.9 cm × 12 cm) and narrowing of the external auditory canal. **(C)** Final CT scan from June 2019, shortly before the patient’s death, demonstrates partial tumor regression with increased calcifications.

On admission, physical examination revealed a firm, fixed, poorly demarcated mass measuring 11 cm × 11 cm in the right temporal region, with overlying skin ulceration and yellow-brown purulent discharge. The mass infiltrated the right orbit, causing ptosis. Hepatomegaly was observed, with the liver palpable 2 cm below the costal margin, while no superficial lymphadenopathy was detected. Laboratory investigations demonstrated pancytopenia (WBC 1.7 × 10^9^/L, HGB 76 g/L, PLT 90 × 10^9^/L), markedly elevated ferritin levels (713.6 ng/mL), and an EBV DNA load of 1.37 × 10^6^ copies/mL, with negative EBV-IgM serology. Liver function tests showed significant abnormalities (AST 224 U/L, LDH 765 U/L), and coagulation studies revealed prolonged APTT (49.5 s) and hypofibrinogenemia (Fg 112 mg/dL). Bone marrow cytology identified classic hemophagocytic activity (**Figure 1**). Computed tomography (CT) imaging revealed a 4.6 cm × 11 cm × 11 cm mass in the right maxillofacial region with destruction of the zygomatic arch, along with hepatosplenomegaly (**Figure 2A**). The patient met five of the HLH-2004 diagnostic criteria: persistent fever, pancytopenia, hyperferritinemia, hemophagocytosis in bone marrow, and hepatosplenomegaly. On December 26, 2018, treatment with dexamethasone (10 mg/m²/day) was initiated to manage HLH.

**Figure 3:**
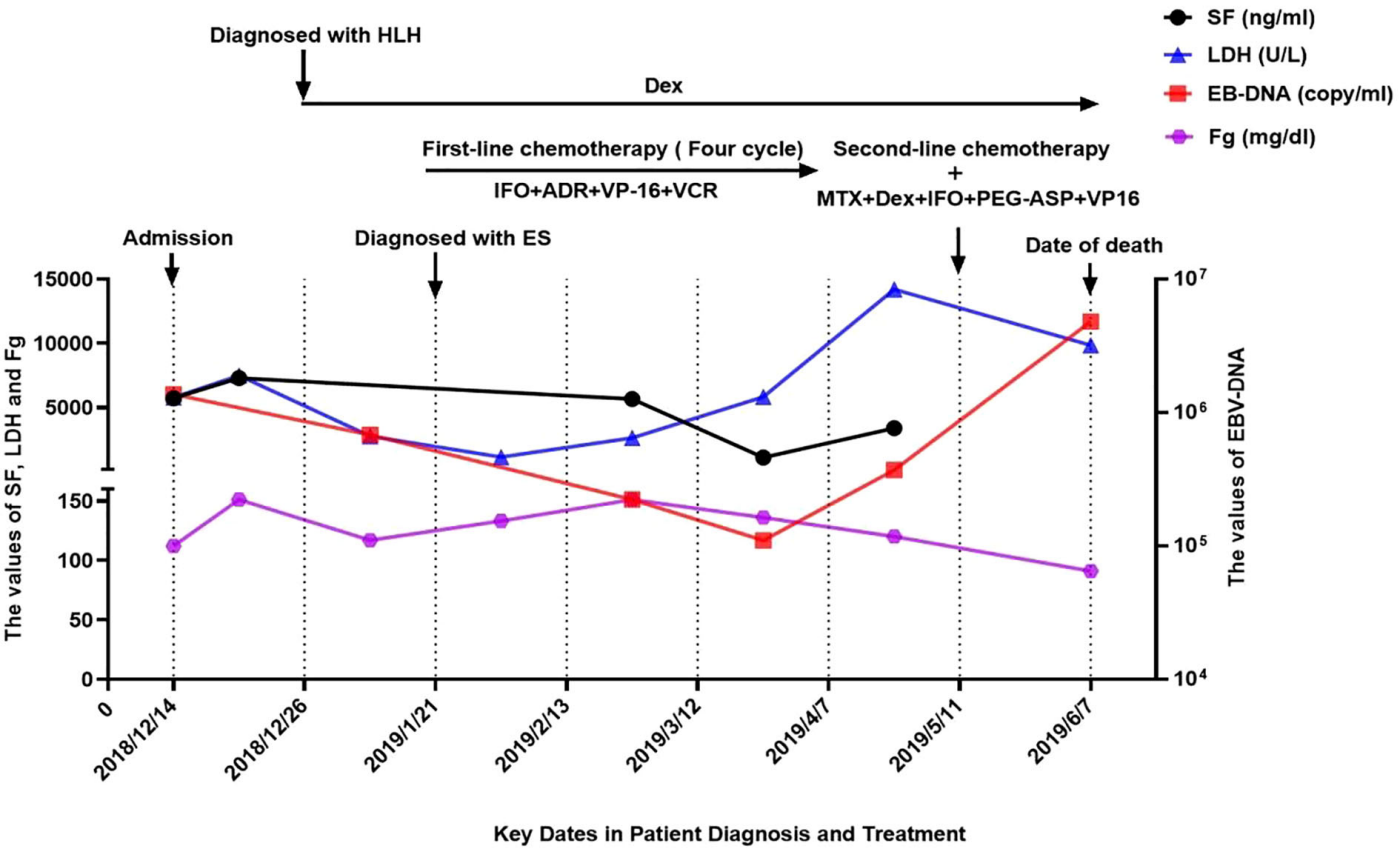
The progression of the disease and the timeline of treatments. LDH, lactate dehydrogenase; SF, serum ferritin; IFO, ifosfamide; ADR, adriamycin; VP-16, etoposide; VCR, vincristine;MTX, methotrexate; DeX, dexamethasone; PEG-ASP, pegylated asparaginase.

In January 2019, pathological biopsy revealed a malignant small round cell tumor with diffuse distribution on H&E staining. Immunohistochemical analysis showed positivity for Vimentin (+) and Fli-1 (+), while markers such as PCK (-), Desmin (-), Myogenin (-), S100 (-), and CD34 (-) were negative. CD99 was partially positive (±), and the Ki-67 proliferation index was approximately 80%. Based on the morphological and immunophenotypic characteristics, a diagnosis of Ewing sarcoma was strongly suggested. However, due to extensive necrosis in the sample, EWSR1-FLI1 fusion gene testing could not be successfully performed. The final diagnosis was confirmed as primary cranial ES complicated by HLH and Epstein-Barr virus (EBV) infection. On January 21, 2019, the patient was initiated on the IVDE chemotherapy regimen, which included ifosfamide (1.8 g/m²/day, days 1-5), doxorubicin (30 mg/m²/day, days 1-2), etoposide (VP-16) (100 mg/m²/day, days 1-5), and vincristine (1.5 mg/m², day 1). This was combined with continuous dexamethasone therapy. During the treatment course, cerebrospinal fluid (CSF) analysis revealed no abnormalities. However, the patient developed a fungal infection, confirmed by histopathological identification of septate hyphae consistent with Aspergillus species. Serum assays showed elevated galactomannan (GM) levels (5.08 GMI) and β-D-glucan (488 pg/mL). Targeted antifungal therapy with voriconazole (6 mg/kg q12h) was initiated. Concurrently, cultures of secretions revealed significant growth of *Staphylococcus epidermidis*, prompting the addition of vancomycin (15 mg/kg q8h) for antimicrobial coverage. Unfortunately, the persistence of HLH and ongoing infection precluded the initiation of radiotherapy and surgical intervention. After four cycles of chemotherapy, follow-up CT scans revealed newly developed metastatic lesions (4.9 cm × 12 cm) and external auditory canal stenosis (**Figure 2B**). Laboratory findings showed progressive deterioration, with lactate dehydrogenase (LDH) levels rising to 14,199 U/L, EBV deoxyribonucleic acid (DNA) load reaching 3.7 × 10^5 copies/mL, and serum ferritin increasing to 3,382 ng/mL. The treatment regimen was adjusted to include high-dose methotrexate (3 g/m²) and pegaspargase (2,500 IU/m²) in combination with the original protocol. By June 2019, imaging studies demonstrated partial tumor regression with increased calcification (**Figure 2C**). However, the patient subsequently developed severe complications, including gastrointestinal bleeding, coagulopathy, and septic shock. The progression of the disease and the timeline of treatments are summarized in **Figure 3**.

## Discussion

ES is a highly aggressive malignant tumor predominantly affecting children and adolescents, with an annual incidence of approximately 2.93 per million (10). It is characterized by rapid proliferation and a propensity for early metastasis. The typical clinical manifestations include localized pain, swelling, and nonspecific systemic symptoms such as fever and anemia (11). Radiologically, ES often presents with lytic bone destruction accompanied by an “onion-skin” periosteal reaction (12). Histopathologically, it is defined by sheets of small round blue cells, with immunohistochemical markers such as CD99 (MIC2 antigen) and FLI-1 being highly characteristic (13). Molecular analysis reveals the EWSR1-FLI1 fusion gene in approximately 85% of cases (14).

ES typically occurs in the pelvis, femur, and axial skeleton. Primary cranial involvement is rare, representing only 3.8% of cases (15). Temporal bone involvement is even more uncommon, with fewer than 40 cases reported globally (16). Here, we present a more rare case of temporal bone ES in a pediatric patient, complicated by HLH and EBV infection. Despite aggressive management, the patient succumbed to gastrointestinal bleeding, septic shock, and multi-organ failure. This report highlights the diagnostic and therapeutic challenges encountered in this case, along with a review of the relevant literature.

The diagnosis of ES is established based on histopathological and molecular. Evaluation of malignant tissue obtained through tumor biopsy. Common biopsy approaches include core needle biopsy (CNB) and open biopsy. Currently, CNB is a widely utilized technique for diagnosing ES (14). The patient initially presented with asymptomatic mass in the right temporal region. Initial fine-needle aspiration biopsy at an external institution suggested cellulitis, but anti-infective therapy was ineffective. Six months later, the diagnosis of ES was confirmed after a second biopsy in our hospital, which incorporated immunohistochemical analysis (Vim+/FLI-1+/Ki67 80%). Regrettably, due to extensive tissue necrosis, the EWSR1-FLI1 fusion gene could not be verified. Notably, this patient had concurrent HLH and EBV infection, while recurrent bacterial and fungal infections significantly impacted both chemotherapy regimen selection and radiotherapy implementation. This case emphasizes that for antibiotic-refractory craniofacial masses, early multidisciplinary consultation (surgery, oncology, pathology) and repeat biopsies are crucial to minimize diagnostic delays, guide therapeutic decision-making, and optimize patient outcomes.

While the HLH-2004 diagnostic criteria remain the gold standard for diagnosing pediatric HLH (3), identifying M-HLH poses significant challenges. First, M-HLH is rare and lacks specific biomarkers. Second, its nonspecific clinical manifestations, such as fever and cytopenia, often overlap with symptoms of the underlying malignancy, increasing the risk of delayed or missed diagnosis (17). Recent studies suggest that certain laboratory parameters may aid in the early recognition of M-HLH. A combination of soluble CD25 (sCD25) > 3900 U/mL and ferritin > 1000 ng/mL has demonstrated a diagnostic sensitivity of 84% and specificity of 81% for M-HLH (18). In the present case, Our patient met HLH diagnostic criteria with persistent fever, pancytopenia, hyperferritinemia, hypofibrinogenemia, and bone marrow hemophagocytosis. although sCD25 levels and NK cell activity were unavailable, the patient exhibited extreme hyperferritinemia (5,713.6 ng/mL), markedly elevated LDH (5,765 U/L), a known marker of malignant associated with poor prognosis (19, 20), and cellulitis-like lesions unresponsive to broad-spectrum antibiotics. These findings prompted further investigation for an underlying malignancy. This case highlights the need for a high suspicion of malignancy in patients with unexplained hyperferritinemia and elevated LDH. Persistent space-occupying lesions unresponsive to antimicrobial therapy should also raise concern.

EBV is a well-known causative agent of M-HLH, particularly in lymphomas (21). EBV-HLH pathogenesis is primarily driven by hypercytokinemia resulting from aberrant activation of macrophages and T/NK cells (22). The main treatment principles for EBV-HLH are suppression of hyperinflammation, elimination of EBV, and replacement of the defective immune system. However, current conventional regimens exhibit limited efficacy against EBV. While rituximab has shown significant clinical utility in EBV-driven HLH cases involving B-cell malignancies, its applicability may be restricted in certain populations (23). In Asian patients, EBV predominantly infects T and NK cells, as the patient was an Asian child with a solid tumor, existing EBV-HLH therapeutic approaches may not be directly applicable. Intriguingly, our findings align with prior studies (24, 25), demonstrating that dynamic monitoring of EBV-DNA levels may serve as a potential biomarker for assessing treatment response in ES. This observation further underscores the need to investigate EBV-directed therapies in ES-associated HLH.

The standardized treatment of ES relies on a multidisciplinary team (MDT) approach, integrating systemic chemotherapy, surgical resection, and radiotherapy (14). Systemic chemotherapy typically follows an alternating VAC/IE regimen, with surgical resection preferred over radiotherapy due to its superior local control (26). However, the complex anatomy of craniofacial ES often limits surgical feasibility, resulting in a 5-year survival rate of less than 50% (15). The coexistence of ES and HLH further complicates management. Immunosuppressive therapy for HLH, such as dexamethasone, may worsen tumor progression. Intensified chemotherapy, on the other hand, increases the risk of HLH-related bone marrow suppression and infections (27). In the present case, the patient showed no improvement in ferritin levels, LDH, or tumor size following initial dexamethasone treatment, suggesting refractory HLH. This required consideration of intensified regimens, such as the DEP protocol, which includes liposomal doxorubicin, etoposide, and methylprednisolone (28). However, the DEP regimen has primarily been studied in adults with lymphoma-associated HLH. For unresectable craniofacial ES, radiotherapy combined with chemotherapy remains a critical strategy for local control (14, 16). However, in this case, rapid disease progression, persistent HLH, and recurrent bacterial/fungal infections associated with the mass, precluded the initiation of radiotherapy.

This case highlights the diagnostic and therapeutic challenges of primary cranial ES complicated by HLH and EBV infection in a pediatric patient. Key clinical insights emerge from this challenging presentation: First, the initial misdiagnosis of antibiotic-resistant craniocerebral masses underscores the importance of early repeat biopsy to prevent diagnostic delays and missed therapeutic windows. Second, the management dilemma involves balancing HLH control against potential tumor progression, particularly when cranial radiotherapy- the preferred local control modality for complex craniofacial ES - must be deferred due to refractory HLH, persistent EBV infection, and recurrent bacterial/fungal infections. Ultimately, despite maximal therapeutic efforts, the patient progressed to fatal multi-organ failure secondary to uncontrolled HLH and superimposed infections.

## Conclusion

This case represents the first reported instance of primary ES of the skull complicated by HLH and EBV infection in a pediatric patient. This case underscores the need for malignancy screening in craniofacial lesions associated with refractory HLH and aberrant EBV activation. Early integration of targeted therapies and radiotherapy should be prioritized to improve local disease control. Multicenter collaborations are crucial for establishing diagnostic and therapeutic consensus for rare and complex clinical conditions. These efforts are necessary to address the challenges associated with current treatment approaches.

## Data Availability

The raw data supporting the conclusions of this article will be made available by the authors, without undue reservation.

## References

[1] Griffin G Shenoi S Hughes GC. Hemophagocytic lymphohistiocytosis: An update on pathogenesis, diagnosis, and therapy. Best Pract Res Clin Rheumatol. (2020) 34:101515. doi: 10.1016/j.berh.2020.101515, PMID: 32387063

[2] Ponnatt TS Lilley CM Mirza KM. Hemophagocytic lymphohistiocytosis. Arch Pathol Lab Med. (2022) 146:507–19. doi: 10.5858/arpa.2020-0802-RA, PMID: 34347856

[3] Henter JI Horne A Aricó M Egeler RM Filipovich AH Imashuku S. HLH-2004: Diagnostic and therapeutic guidelines for hemophagocytic lymphohistiocytosis. Pediatr Blood Cancer. (2007) 48:124–31. doi: 10.1002/pbc.21039, PMID: 16937360

[4] Campo M Berliner N. Hemophagocytic lymphohistiocytosis in adults. Hematol Oncol Clin North Am. (2015) 29:915–25. doi: 10.1016/j.hoc.2015.06.009, PMID: 26461151

[5] Monti M Marconi G Ambrosini-Spaltro A Gallio C Ghini V Esposito L. Hemophagocytic lymphohistiocytosis in gastric cancer: A rare syndrome for the oncologist. Case report and brief review. Front Oncol. (2023) 13:1010561. doi: 10.3389/fonc.2023.1010561, PMID: 36845741 PMC9945267

[6] Kumar V Eulitt PJ Bermudez A Khagi S. Hemophagocytic lymphohistiocytosis in a patient with glioblastoma: a case report. CNS Oncol. (2019) 8:Cns45. doi: 10.2217/cns-2019-0013, PMID: 31777271 PMC6912850

[7] Rajapakse P Shrestha SD Bakirhan K. Hemophagocytic lymphohistiocytosis secondary to prostatic adenocarcinoma. Cureus. (2021) 13:e12798. doi: 10.7759/cureus.12798, PMID: 33628667 PMC7894226

[8] Guo T Liu Z Chen Y Cheng Y He K Lin X. Hemophagocytic lymphohistiocytosis during treatment of intracranial multifocal germinoma: a case report and literature review. Front Oncol. (2024) 14:1264926. doi: 10.3389/fonc.2024.1264926, PMID: 38532931 PMC10963405

[9] Kalmuk J Puchalla J Feng G Giri A Kaczmar J. Pembrolizumab-induced Hemophagocytic Lymphohistiocytosis: an immunotherapeutic challenge. Cancers Head Neck. (2020) 5:3. doi: 10.1186/s41199-020-0050-3, PMID: 32025343 PMC6996173

[10] Grünewald TGP Cidre-Aranaz F Surdez D Tomazou EM de Álava E Kovar H. Ewing sarcoma. Nat Rev Dis Primers. (2018) 4:5. doi: 10.1038/s41572-018-0003-x, PMID: 29977059

[11] Balamuth NJ Womer RB. Ewing’s sarcoma. Lancet Oncol. (2010) 11:184–92. doi: 10.1016/S1470-2045(09)70286-4, PMID: 20152770

[12] Rana RS Wu JS Eisenberg RL. Periosteal reaction. AJR Am J Roentgenol. (2009) 193:W259–72. doi: 10.2214/AJR.09.3300, PMID: 19770293

[13] Marcilla D Machado I Grünewald TGP Llombart-Bosch A de Álava E. (Immuno)histological analysis of ewing sarcoma. Methods Mol Biol. (2021) 2226:49–64. doi: 10.1007/978-1-0716-1020-6_5, PMID: 33326093

[14] Zöllner SK Amatruda JF. Ewing sarcoma-diagnosis, treatment, clinical challenges and future perspectives. J Clin Med. (2021) 10:1685. doi: 10.3390/jcm10081685, PMID: 33919988 PMC8071040

[15] Nair D Thomas L Patil V. Ewing’s sarcoma of the head and neck: differential diagnosis, treatment and outcomes. Curr Opin Otolaryngol Head Neck Surg. (2025) 33:85–91. doi: 10.1097/MOO.0000000000001032, PMID: 39752208

[16] Vishnoi JR Kumar V Srivastava K Misra S. Primary Ewing’s sarcoma of the temporal bone: a rare entity and review of the literature. BMJ Case Rep. (2019) 12:e230768. doi: 10.1136/bcr-2019-230768, PMID: 31645395 PMC6827776

[17] Zoref-Lorenz A Witzig TE. Malignancy-associated HLH: mechanisms, diagnosis, and treatment of a severe hyperinflammatory syndrome. Leuk Lymphoma. (2024) p:1–9. doi: 10.1080/10428194.2024.2436037, PMID: 39656557

[18] Zoref-Lorenz A Murakami J. An improved index for diagnosis and mortality prediction in Malignancy-associated hemophagocytic lymphohistiocytosis. Blood. (2022) 139:1098–110. doi: 10.1182/blood.2021012764, PMID: 34780598 PMC8854682

[19] Li F Li P Zhang R Yang G Ji D Huang X. Identification of clinical features of lymphoma-associated hemophagocytic syndrome (LAHS): an analysis of 69 patients with hemophagocytic syndrome from a single-center in central region of China. Med Oncol. (2014) 31:902. doi: 10.1007/s12032-014-0902-y, PMID: 24610542

[20] Del Baldo G Abbas R. The prognostic role of the C-reactive protein and serum lactate dehydrogenase in a pediatric series of bone ewing sarcoma. Cancers (Basel). (2022) 14:3064. doi: 10.3390/cancers14133064, PMID: 35804835 PMC9264769

[21] El-Mallawany NK Curry CV. Hemophagocytic lymphohistiocytosis and Epstein-Barr virus: a complex relationship with diverse origins, expression and outcomes. (2022) 196:31–44. doi: 10.1111/bjh.17638, PMID: 34169507

[22] Parvaneh N Filipovich AH Borkhardt A. Primary immunodeficiencies predisposed to Epstein-Barr virus-driven hematological diseases. Br J Hematol. (2013) 162:573–86. doi: 10.1111/bjh.12422, PMID: 23758097

[23] Meng GQ Wang JS Wang YN Wei N Wang Z. Rituximab-containing immuno-chemotherapy regimens are effective for the elimination of EBV for EBV-HLH with only and mainly B lymphocytes of EBV infection. Int Immunopharmacol. (2021) 96:107606., PMID: 33826999 10.1016/j.intimp.2021.107606

[24] Yang JH Sun XS Xiao BB Liu LT Guo SS Liang JD. Subdivision of de-novo metastatic nasopharyngeal carcinoma based on tumor burden and pretreatment EBV DNA for therapeutic guidance of locoregional radiotherapy. BMC Cancer. (2021) 21:534. doi: 10.1186/s12885-021-08246-0, PMID: 33975558 PMC8111972

[25] Zhang W Peng Y Qiu Y Cheng L Yin Y Li Y. Clinical significance and different strategies for re-elevation of plasma EBV-DNA during treatment in pediatric EBV-associated hemophagocytic lymphohistiocytosis. J Pediatr (Rio J). (2024) 100:505–11. doi: 10.1016/j.jped.2024.03.006, PMID: 38604242 PMC11361887

[26] Ladenstein R Pötschger U Le Deley MC Whelan J Paulussen M Oberlin O. Primary disseminated multifocal Ewing sarcoma: results of the Euro-EWING 99 trial. J Clin Oncol. (2010) 28:3284–91. doi: 10.1200/JCO.2009.22.9864, PMID: 20547982

[27] Otrock ZK Eby CS. Clinical characteristics, prognostic factors, and outcomes of adult patients with hemophagocytic lymphohistiocytosis. Am J Hematol. (2015) 90:220–4. doi: 10.1002/ajh.23911, PMID: 25469675

[28] Zhao Y Li Z. L-DEP regimen salvage therapy for pediatric patients with refractory Epstein-Barr virus-associated hemophagocytic lymphohistiocytosis. Br J Haematol. (2020) 191:453–9. doi: 10.1111/bjh.16861, PMID: 32525580

